# Supplementary material for: The Maize Gene ZmGLYI-8 Confers Salt and Drought Tolerance in Transgenic Arabidopsis Plants
Source: Int J Mol Sci. 2024 Oct 11;25(20):10937. doi: 10.3390/ijms252010937 (PMC11507017; doi:10.3390/ijms252010937)
Supplement: Supplementary file 1 [file ijms-25-10937-s001.zip › ijms-3219784-supplementary.pdf]

Table S1. Primers used in this study.

Primer	Sequence(5'-3')
ZmGLYI-8/F	ATCTAGAGCCACGATGGCGCGCCT
ZmGLYI-8/R	AGTCGACCTCATTCTTCCAACTCT
ZmGLYI-8/2R	ACTCGAGTTCTTCCAACTCTTTCAG
ZmGLYI-8/2F	GAATTCTCATCGCTCCACCTCGCC
qZmGLYI-8/F	AGGAGGAACAGTCACAAGG
qZmGLYI-8/R	ACAAAGGCTCGGGAGTAG
Actin/F	GGGATTGCCGATCGTATGAG
Actin/R	GAGCCACCGATCCAGACACT

ZmGLYI-8/F and ZmGLYI-8/R for *ZmGLYI-8* full-length sequence amplification.
ZmGLYI-8/F and ZmGLYI-8/2R were used to construct prokaryotic expression vectors.
ZmGLYI-8/2F and ZmGLYI-8/R were used to construct subcellular localization vectors.
ZmGLYI-8/F and ZmGLYI-8/R for qRT-PCR analysis.
Actin/F and Actin/R for the endogenous control.

```
                10        20        30        40        50        60        70        80        90
1        ATGGCGCGCCTGCTCATCCCCCTCCCCTTCGCCGCCGCCTCTGCCTCATCGCTCCACCTCGCCGTCTCCCGCCTTCCTCTCGCCGCGGTC
1         M  A  R  L  L  I  P  L  P  F  A  A  A  S  A  S  S  L  H  L  A  V  S  R  L  P  L  A  A  V

               100       110       120       130       140       150       160       170       180
91       TCCGCCGCGCGTCGCGAGTGTCTCTTTGGGGGAAGGGTGGTAGGAGGAGTGGTGAGGGCGCCCGCGAGGCTTTCTAAGCGCGGGTTGTGC
31        S  A  A  R  R  E  C  L  F  G  G  R  V  V  G  G  V  V  R  A  P  A  R  L  S  K  R  G  L  C

               190       200       210       220       230       240       250       260       270
181      GCCGGCGCAGAGGCTGGCGACTCGGCCGTCACGGTGGAGGCCATGGAATGGGTCAAGAAGGACAGGAGGCGCCTGCTCCACGTCGTCTAC
61        A  G  A  E  A  G  D  S  A  V  T  V  E  A  M  E  W  V  K  K  D  R  R  R  L  L  H  V  V  Y

               280       290       300       310       320       330       340       350       360
271      CGCGTCGGGGACCTCGACAAGACGATCAAGTTCTACACGGAGTGCCTGGGCATGAAACTGTTGAGGAAGAGGGACATTCCCGAGGAGAGG
91        R  V  G  D  L  D  K  T  I  K  F  Y  T  E  C  L  G  M  K  L  L  R  K  R  D  I  P  E  E  R

               370       380       390       400       410       420       430       440       450
361      TACACCAATGCCTTTCTGGGGTACGGGCCTGAGGATTCACATTTTGTTGTGGAGCTCACTTACAATTATGGTGTGGAGAGCTATAACATC
121       Y  T  N  A  F  L  G  Y  G  P  E  D  S  H  F  V  V  E  L  T  Y  N  Y  G  V  E  S  Y  N  I

               460       470       480       490       500       510       520       530       540
451      GGGACTGGTTTTGGCCACTTTGGAATTGCTGTTGAGGATGTTGCAAAAACAGTGGAACTTATTAAAGCAAAAGGAGGAACAGTCACAAGG
151       G  T  G  F  G  H  F  G  I  A  V  E  D  V  A  K  T  V  E  L  I  K  A  K  G  G  T  V  T  R

               550       560       570       580       590       600       610       620       630
541      GAGCCAGGCCCTGTCAAAGGTGGGAAATCAGTAATTGCCTTTATTGAGGATCCTGATGGTTACAAGTTTGAGCTTATAGAAAGAGGGCCT
181       E  P  G  P  V  K  G  G  K  S  V  I  A  F  I  E  D  P  D  G  Y  K  F  E  L  I  E  R  G  P

               640       650       660       670       680       690       700       710       720
631      ACTCCCGAGCCTTTGTGCCAGGTAATGCTTCGAGTGGGAGATCTTGATCGTGCTATAAATTTCTATGAGAAGGCATTTGGCATGGAACTT
211       T  P  E  P  L  C  Q  V  M  L  R  V  G  D  L  D  R  A  I  N  F  Y  E  K  A  F  G  M  E  L

               730       740       750       760       770       780       790       800       810
721      CTTCGCAAGCGAGACAATTCTGAGTACAAGTATACGATTGCGATGATGGGATATGGTCCAGAAGACAAAAATGCTGTATTGGAGTTGACC
241       L  R  K  R  D  N  S  E  Y  K  Y  T  I  A  M  M  G  Y  G  P  E  D  K  N  A  V  L  E  L  T

               820       830       840       850       860       870       880       890       900
811      TACAACTATGGGGTGAAGGAATATGATAAGGGAAATGCTTATGCACAGATTGCTATTAGTACTGATGATGTCTACAAAACTGCGGAAGCA
271       Y  N  Y  G  V  K  E  Y  D  K  G  N  A  Y  A  Q  I  A  I  S  T  D  D  V  Y  K  T  A  E  A

               910       920       930       940       950       960       970       980       990
901      ATTAGAGTAAATGGTGGACAAATTACTCGTGAACCTGGCCCATTACCTGGCATCACCACCAAGATAACTGCATGCACAGATCCAGATGGC
301       I  R  V  N  G  G  Q  I  T  R  E  P  G  P  L  P  G  I  T  T  K  I  T  A  C  T  D  P  D  G

              1000      1010      1020      1030      1040
991      TGGAAAACAGTGTTTGTCGATAACATAGATTTTCTGAAAGAGTTGGAAGAATGA
331       W  K  T  V  F  V  D  N  I  D  F  L  K  E  L  E  E  *
```

Supplemental Figure S1. Nucleotide sequence and amino acid sequence of *ZmGLY□-8*.

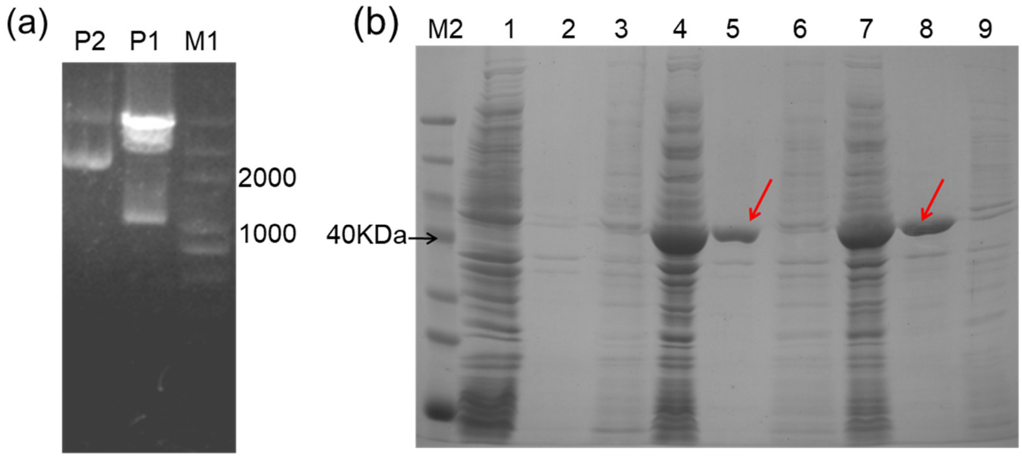

Supplemental Figure S2. □nduced expression of *ZmGLY□-8* in *E.coli* Rosetta. (a) □dentification of recombinant vector pET-30a- *ZmGLY□-8* by double enzyme digestion. M1, DNA marker 5000; P1, pET-30a-*ZmGLY□-8* recombinant plasmid DNA; P2, digestion of pET-30a-*ZmGLY□-8* DNA. (b) ZmGLY□-8 protein was detected by SDS-PAGE in Rosetta containing pET-30a-*ZmGLY□-8*. M, Protein Marker; 1-3, the total protein, precipitation and supernatant of empty plasmid pET30a in *E.coli* induced by 0.1 mmol/L □PTG; 4-6 and 7-9, the total protein, precipitation and supernatant of pET-30a- *ZmGLY□-8* induced by 0.5 mmol/L □PTG under 28 °Cand 37 °C. The *ZmGLY□-8* protein is marked with a red arrow.

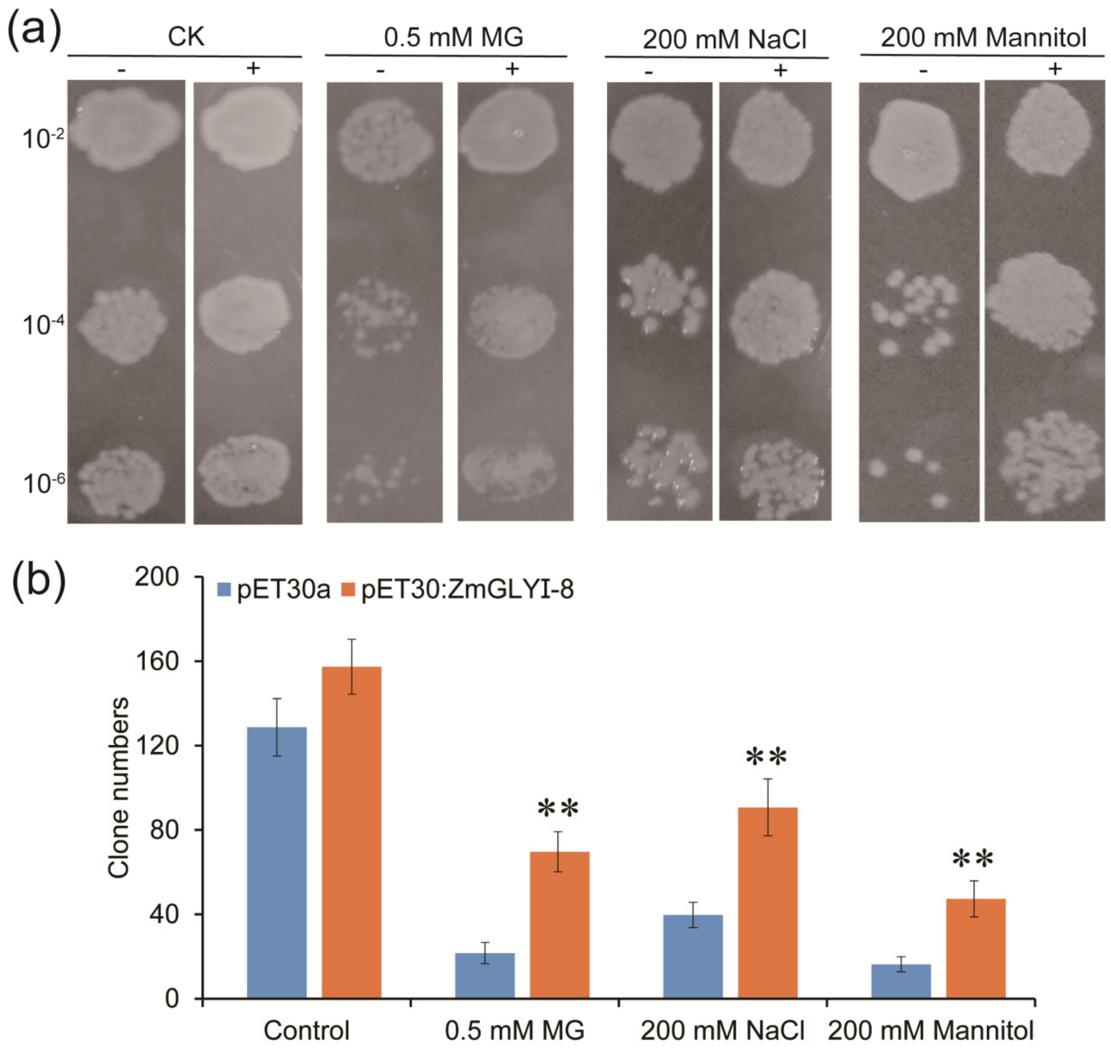

Supplemental Figure S3. Analysis of *ZmGLY□8* prokaryotic expression. (a) Droplet plate experiment of the recombinant strain pET-30a-*ZmGLY□8* and the control strain pET-30a under various abiotic stresses (200 mM mannitol, 200 mM NaCl and 0.5 mM MG). (b) Number of colonies of the recombinant strain pET-30a-*ZmGLY□8* and the control strain (pET-30a) under various abiotic stresses (200 mM mannitol, 200 mM NaCl and 0.5 mM MG).

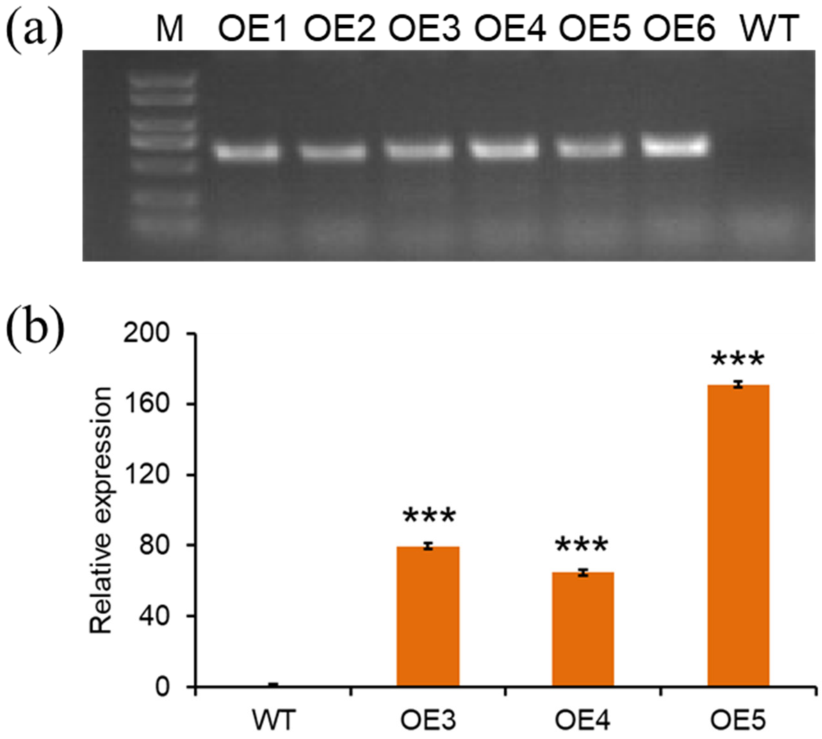

Supplemental Figure S4. PCR amplification (a) and qRT-PCR (b) analysis of *ZmGLY□8* transgenic *Arabidopsis* lines. OE1-OE6: transgenic *Arabidopsis* lines. WT: wild-type. ***$p < 0.001$.
